# Artificial intelligence for precision medicine in autoimmune liver disease

**DOI:** 10.3389/fimmu.2022.966329

**Published:** 2022-11-11

**Authors:** Alessio Gerussi, Miki Scaravaglio, Laura Cristoferi, Damiano Verda, Chiara Milani, Elisabetta De Bernardi, Davide Ippolito, Rosanna Asselta, Pietro Invernizzi, Jakob Nikolas Kather, Marco Carbone

**Affiliations:** ^1^ Division of Gastroenterology, Center for Autoimmune Liver Diseases, Department of Medicine and Surgery, University of Milano-Bicocca, Monza, Italy; ^2^ European Reference Network on Hepatological Diseases (ERN RARE-LIVER), San Gerardo Hospital, Monza, Italy; ^3^ Bicocca Bioinformatics Biostatistics and Bioimaging Centre - B4, School of Medicine and Surgery, University of Milano-Bicocca, Monza, Italy; ^4^ Rulex Inc., Newton, MA, United States; ^5^ Department of Medicine and Surgery and Tecnomed Foundation, University of Milano - Bicocca, Monza, Italy; ^6^ Department of Radiology, San Gerardo Hospital, Monza, Italy; ^7^ Humanitas Clinical and Research Center, Rozzano, Milan, Italy; ^8^ Department of Biomedical Sciences, Humanitas University, Pieve Emanuele, Milan, Italy; ^9^ Department of Medicine III, University Hospital RWTH Aachen, Aachen, Germany; ^10^ Else Kroener Fresenius Center for Digital Health, Medical Faculty Carl Gustav Carus, Technical University Dresden, Dresden, Germany

**Keywords:** machine learning, genomics, radiomics, digital pathology, autoimmunity, deep learning, whole-slide digital image analysis, population genetics

## Abstract

Autoimmune liver diseases (AiLDs) are rare autoimmune conditions of the liver and the biliary tree with unknown etiology and limited treatment options. AiLDs are inherently characterized by a high degree of complexity, which poses great challenges in understanding their etiopathogenesis, developing novel biomarkers and risk-stratification tools, and, eventually, generating new drugs. Artificial intelligence (AI) is considered one of the best candidates to support researchers and clinicians in making sense of biological complexity. In this review, we offer a primer on AI and machine learning for clinicians, and discuss recent available literature on its applications in medicine and more specifically how it can help to tackle major unmet needs in AiLDs.

## 1 Introduction

Autoimmune liver diseases (AiLDs) are chronic diseases affecting the liver and the biliary tract, with a putative autoimmune pathogenesis, and include autoimmune hepatitis (AIH) (1), primary biliary cholangitis (PBC) (2), and primary sclerosing cholangitis (PSC) (3). The combination of low prevalence, unknown etiology, and high degree of heterogeneity among patients fulfilling the same diagnostic criteria have hitherto hindered the development of drugs, especially for PSC and AIH.

**Figure 1:**
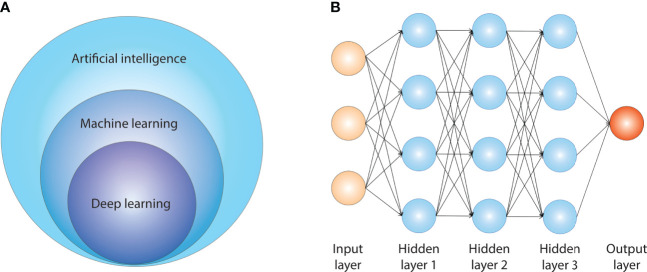
Artificial intelligence and its sub-fields.

Nonetheless, high-throughput DNA and RNA sequencing technologies, digital pathology, and digital radiology are also progressively reaching this neglected field. A large amount of experimental and clinical data are increasingly available in the field (4), which requires dedicated analytical pipelines that are able to deal with big data. Artificial intelligence (AI) is a broad scientific field including many sub-specialties. **Figure 1A** summarizes the relationship between AI, machine learning (ML), and deep learning (DL). AI comprises several sub-fields, and the most important ones for medical applications are ML and DL. ML algorithms create models that learn from sample data (training data) and are then able to make inference/predictions on new data without being explicitly programmed for this scope. Among the others, ML is of particular interest for the biomedical field, since it can recognize patterns within data and leverage them to generate new biological knowledge. DL is a sub-field of ML that uses multiple layers of information for extraction of features from raw inputs. This type of AI is particularly well-suited for image processing.

This review aims to provide an introduction to AI for clinicians and to outline the current evidence about AI in medicine and the foreseeable applications in the field of AiLDs.

## 2 Artificial intelligence: Working definitions and its growing role in the biomedical field

In 1959, Artur L. Samuel, a computer scientist, firstly introduced the expression “machine learning” in his seminal paper focusing on how a machine could learn the game of checkers (5). In line with Samuel’s definition, ML could be defined as that sub-field of AI in which computers are not explicitly programmed by experts but rather learn from experience (6). For instance, this could be the case of a process that analyzes historical data concerning the time needed to recover from a disease, and it is later used to predict the recovery time for new patients with the same disease. In this example, a set of *labeled* data is available, e.g., the number of days needed to recover; this value is the value of the *target* (alternatively referred to as the *output*) of the problem at hand, and it is known for a set of the patients tracked in the historical record. When the value of the output is known and labeled, the problem falls into the area of *supervised learning*. When the output of interest is a *quantity* (e.g., the number of days needed to recover), the problem is referred to as a *regression*. On the contrary, if the *output* represents a *quality* (a binary one: disease status versus healthy status), the problem would be defined as a *classification*.

In other cases, the set of *labeled* data is not available and the macro-category is called *unsupervised learning*. For example, unsupervised learning may refer to the task of splitting a set of patients into homogeneous subgroups with respect to a set of features (*clustering*) or rather to determine which diagnostic factors are correlated to each other (*association mining)*. In these cases, there is no known, pre-defined and pre-labeled target of interest.

All these tasks can potentially be addressed with a statistical approach. The main difference between statistics and ML is that ML does not require to make any assumptions concerning the statistical distribution of the considered features (7). Conversely, the outcome of a statistical pipeline relies on (and benefits from) the knowledge about the underlying distribution of the considered population, as well as the statistical properties of the chosen estimator. This requirement makes the statistical approach less affordable in the case of high-dimensional data, as it further increases complexity.

Conversely, considering that ML approaches cannot be compared against any reference distribution for evaluation, it is harder to assess their performance. A common procedure to deal with this issue consists in comparing the model predictions against the data themselves. More specifically, a subset of data (for instance, a subset of patients whose data are available in the historical record) is used to *train* the model, while another part is not supplied to the training pipeline. In ML terminology, the former part is usually referred to as *training set*, while the latter is commonly denoted as *test set*. After having completed the modeling phase on the *training set*, the model itself is applied on the *test set*; in other words, the model will make predictions on a new and unknown subset of data, which simulates the foreseeable new data on which it will be applied in the future. Coming back to the first example about days to recover from a disease, the *test set* will involve patients not considered in the modeling phase on training. More complex and sophisticated scenarios also involve the introduction of a third dataset, referred to as the *validation* dataset; in this case, there is a dataset for *training*, a dataset for *hyperparameter tuning* (tuning of model parameters) on top of training, and another one for *performance evaluation*.

External validation should be performed for rigorous evaluation of the algorithm performance. In fact, deficiency and biases present in the training dataset may appear in external data that are either closer to the ideal target population or representative of minority populations. In this way, the risk of biased performance estimation is reduced and more consistent and robust conclusions can be drawn (8).

Evaluating, for instance, the *root mean squared error* (or, in the case of a *classification*, other indicators such as *empirical accuracy* or *area under the curve*) allows to understand how much the extracted model is effective on previously unseen data. When there is a strikingly good performance in the *training set*, but with a poor one in the *test set*, the model is *overfitting* data: in other words, it is too focused on replicating training data and is not able to generalize its predictive power.

Among the different ML available models, some of the well-known approaches are artificial neural networks (9) and support vector machines (10). In the latest couple of decades, neural networks and especially multi-layered ones became even more popular, being at the core of DL approaches. DL refers to a pipeline in which the learning process is modularized: the first layer of modeling can be considered as in charge of learning features that will be used by the following layer, enabling the final one to provide a prediction (11). This has been shown to be particularly promising in the field of high-dimensional, *unstructured data*, such as documents or images (12). **Figure 1B** shows the basic architecture of an artificial neural network. DL algorithms have a more sophisticated and complex architecture than non-DL ML algorithms, which include many more free parameters, providing a higher degree of flexibility. The core of DL algorithms is usually the artificial neural network, which is constituted of several nodes called artificial neurons. The output of a neuron is a non-linear computation of the sum of its inputs.

Overall, AI and its sub-fields ML and DL offer a wide range of tools for data mining and predictions, which represent a great opportunity to advance the biomedical field. Multiple examples of AI applications have been produced over the last years in several fields, such as pathology, radiology, dermatology, and endoscopy, to name a few (13). Analyses of images derived from tissue specimens (14), radiological exams (15), and pictures of skin lesions (16) or captured from videos of endoscopic procedures (17) are examples of input data that have been analyzed through different types of neural network algorithms. In addition, clinical data either extracted from multicenter collaborative efforts or derived from electronic health records (EHRs) have also been analyzed with ML software to understand whether the AI-based models were better than available diagnostic and/or prognostic scores (18) or to identify subgroups of individuals at different disease course (19, 20). Neural networks have also shown promising results in several fields of genomics (21); particularly interesting are also approaches that integrate genomic data (most commonly common variants from genome-wide association studies) with images, with the aim to match image-derived markers with gene signatures (15).

## 3 Applications in autoimmune liver diseases

### 3.1 Digital and computational pathology

#### 3.1.1 Aims and applications

Histopathology slides intrinsically hold a large amount of information and data, which have been largely underutilized in the past. The transformation of these analogic data in digital file formats is the core of “digital pathology” (22). The whole slide imaging (WSI) technique implies the digitalization of the whole histological section *via* a digital scanner, and has been progressively becoming more available and widespread.

The revolution carried forward by ML, particularly in its sub-domain of DL, is fostering the development of an associated new field called “computational pathology” (23). This sub-field regards all the processes involved in extracting and handling data present in digital slides to generate valuable information for clinical and research purposes. Overall, computational pathology may be capable to provide solutions to several issues in modern medicine. Schematically, we can enucleate several applications: in the diagnostic area, it favors automation, supports the pathologist, and enhances telemedicine; in the research area, it improves the understanding of the pathogenesis at the cellular and tissue levels, prognostication, and risk stratification.

In hepatology, and in rare disorders like AiLDs, histological evaluation of liver tissue may play an important role in the diagnostic workout. Moreover, it may offer semiquantitative/quantitative prognostic information and supports choice of therapies. Therefore, hepatology represents an appropriate field for WSI and computational pathology (24).

#### 3.1.2 Whole slide imaging technology and deep learning

The first step in WSI technology is the use of the appropriate technology for image acquisition, i.e., an image scanner for digital image acquisition. The second part of the process is based on the view and analysis of the image through a dedicated software (25).

The most common method to generate images is tiling, i.e., acquiring the original slide as tiles, although a linear scanning system can also be used. The final image is the result of merging of each tile or line scan by the software. DL algorithms extract parameters of interest from the scans by using labels corresponding to predefined categories. They can obviously be used also through an unsupervised manner for clustering or other data grouping strategies.

It is evident that WSI technology coupled with DL could work in a much more automated and efficient way than current standards, and can be of help for clinical practice (26). Arguments in favor of this statement are the improved reproducibility and speed of the diagnostic process together with the reduction of workload for clinicians (26). By reducing the need for repetitive work, pathologists can focus on those tasks that require specific skills like the integration of the available information to produce accurate diagnostic hypotheses and communicate with clinicians. Before translation into clinical practice, it is essential to validate WSI performance compared to standard procedures; few studies are available, but data seem promising (27). Most of the published studies in the hepatological field include only WSI without subsequent DL applications, and are mainly focused on exploring the added value of WSI in terms of better inter-observer concordance. In fact, the digitalization allows better sharing and annotation of tissue slides, making easier collaboration among pathologists (22).

Among the studies that went beyond pure assessment of reproducibility of the diagnostic process, a recent and elegant work from Cheng et al. has shown the excellent accuracy of a DL algorithm applied to images derived from formalin-fixed, paraffin-embedded surgical resections and biopsy specimens of nodular lesions of the liver. On WSI, a pathologist outlined ROIs generating hundreds of thousands patches for further analysis. Several testing sets were available for independent external validation. Three DL models were evaluated, with AUC always > 0.90 in external validation datasets. Of note, to avoid batch effects and increase generalizability, data for external validation were collected from three different hospitals. To us, the core elements of this work are a solid methodology, the involvement of expert pathologists, and the use of a large training set together with heterogeneous validation sets taken from different centers (28).

Another important study from China has shown the capacity of DL applied to WSI derived from hepatocellular carcinoma (HCC) to discriminate the type of tissue and to detect new prognostic biomarkers. In this case, on top of data coming from a single Chinese cohort, the investigators have also leveraged the Cancer Genome Atlas (TCGA) to finely map the identified histological patterns and correlate them with tumor immune infiltrates and gene mutations. Of note, to take into account the different staining protocols between the training cohort and TCGA, they used a specific DL algorithm for standardization. This work represents a remarkable example of a pipeline that goes from slice preparation to the development of a risk score associated to histological patterns identified within the tumor, showing the ability of this score to stratify patients in higher- and lower-risk groups and to correlate score values with histological patterns and tumor-associated immune cells (29).

More relevant to the field of AiLD are applications of WSI and DL to inflammatory diseases such as ulcerative colitis (UC). In a recent landmark paper showing data from an international multicenter consortium, investigators have created a new histological score for UC, which correlates with endoscopic findings and holds prognostic value. To generate the score, colonic biopsies were used and a group of expert pathologists annotated the slides by using a variety of histological scoring schemes already available. After a Delphi consensus among pathologists, the neutrophil infiltrate was deemed as the key element of disease activity and clinical outcome; importantly, the new score was created by using standard statistical methods. Subsequently, the DL algorithm was trained to learn how to identify neutrophil infiltrates within images and to differentiate between quiescent and active disease, achieving 86% accuracy. In our opinion, the interesting aspect of this study is the blended approach between classical and standardized statistical approaches (development of scores and survival analysis) together with DL (17).

WSI offers a more quantitative approach for assessment of liver fibrosis and steatosis, and novel data are available for HCC and transplant pathology (22). A dedicated review of the most recent applications of WSI technology to liver diseases is reported below (22). No specific data are available for AiLD at the time of writing.

#### 3.1.3 Methodological hurdles and limitations

The main limitations to the implementation of AI-based technologies to digital pathology are the lack of universal standards for data formatting, as compared to radiology, where the Digital Imaging and Communications in Medicine (DICOM) format is already the standard (6). The current trend in the field is to use AI to digitalize these processes and reduce arbitrariness and low agreement among pathologists (30).

In addition, data quality is essential; in fact, histological slides comprise a highly heterogeneous information. Staining, thickness of the section, and presence of artifacts are known factors influencing the model performance in DL-based diagnostic models (31). The development and optimization of staining methods to enhance the contrast of biological components has been a goal for decades. Yet, inconsistencies and artifacts are still generated despite technical efforts to improve specimen preparation. This issue represents an obstacle for digital pathology, since a batch effect can be introduced when analyzing altogether samples either from different institutions or from the same institution but retrieved at different time points (32). Novel strategies have been put in practice to overcome this hurdle by means of unsupervised methods based on color normalization and adversarial adaptation (33). Convolutional neural networks (a class of artificial neural network commonly applied for image analysis) are used to learn properties present in the source domain and then apply them on the target domain without any supervised labeling (33, 34). Several specific strategies for prevention of the artifact-driven loss of performance are currently under investigation (31).

Another important limitation of current pipelines that is particularly relevant for the field of AiLD is their exclusive reliance on hematoxylin–eosin stainings. Despite the validated role of CK7 and orcein stainings in the differential diagnosis and staging of PBC (35) and PSC (36), to our knowledge, there are no published computational pathology pipelines that have been trained on this type of images.

Overall, there are still some methodological limitations for the full implementation of digital pathology as a research tool and for its incorporation in the current clinical workflow; yet, there is active research aiming at addressing them.

#### 3.1.4 Potential applications in AiLD

Liver biopsy has a pivotal role in the diagnostic and prognostic process of AiLDs. It is essential for the diagnosis of AIH and holds value in atypical cases of PBC and PSC; for all the three conditions, it provides a rich amount of information that can assist prognostication and guide treatment. While for PBC the pediatric onset is exceptional, AIH and PSC can arise at pediatric age, with features and disease course different to the adult onset.

Computational pathology has several promising applications in the field of AiLDs. As the pathogenesis of these diseases is still obscure, the use of AI-assisted methods may aid in discovering pathogenetic clues for further investigation. Computational pathology may also assist in identifying core histological features of AiLDs that are still missing, and provide a more standardized approach for differential diagnosis (e.g., discriminating between pure PBC or AIH and variant syndromes). An even more promising application is for prognosis modeling and risk stratification.

Nevertheless, there are some disease-specific traits that can influence AI implementation. In AIH, liver biopsy is the cornerstone of the diagnosis, and the available scoring systems unlikely lead to definite AIH diagnosis without histological evaluation. In addition, histology provides useful information on disease activity and stage (37). Histological diagnostic criteria evolve over time, and AIH makes no exception. The combination of interface hepatitis and a predominantly periportal lympho-plasmacellular infiltrate is nearly always present in AIH, but it is not pathognomonic (38). The recent revision of histological criteria for the diagnosis of AIH has recognized centrilobular injury, together with central perivenulitis and necrosis, as being part of the histological spectrum of acute severe AIH (39). However, qualitative or semi-quantitative information derived from liver histology of patients with AIH is currently insufficient to accurately depict its heterogeneous histological phenotypes and to predict treatment response and/or relapse. Moreover, the differential diagnosis with other forms of hepatitis, especially acute hepatitis or drug-induced liver injury, remains extremely difficult. A foreseeable goal would be to have quantitative metrics of subtle processes such as the extension of lympho-plasmacellular infiltrates within the periportal tract, lobular necrosis, and other pathological processes that are frequently observed in AIH, to develop a more reliable diagnostic and prognosis prediction approach. It appears evident that AIH would hugely benefit from the application of digital pathology because the digital analysis of histological slides could offer a standardized approach to its diagnosis and could help to identify features that are inherently proper of AIH rather than drug-induced liver injury or other AIH-mimics (40). ML is required to deal with the large amount of information that would be obtained after extraction of quantitative metrics from digital slides (feature selection).

For risk stratification in AIH, unsupervised ML approaches could be of interest to identify prognostic biomarkers of disease activity that can predict biochemical remission, when liver biopsy is performed at diagnosis, or relapse, if the histological assessment is performed before treatment withdrawal. Unsupervised learning could shed a light on morphological features peculiar of sub-phenotypes of AIH that are currently invisible to the human eye, highlighting populations of cells or morphological patterns that are not considered part of the histological spectrum of the disease (41–44). Unsupervised learning techniques are commonly used to analyze and interpret single-cell RNA sequencing data; for instance, Liu et al. have recently shown that single-cell profiling of immune transcriptomes of skin samples from patients with different inflammatory skin disorders can differentiate among different conditions by identifying, in a unbiased manner, gene expression signatures (41). On a similar note, the analysis of approximately 200 million nuclei from digitized slides of 117 patients affected by glioblastoma was able to derive three disease clusters with different nuclei morphology and specific associations with gene signatures (44).

Even though only supervised learning approaches were employed, in another landmark paper from Stanford University, it was shown that the use of a computational pathology tool that automatically generates quantitative features can pinpoint structures that had not been called in action as prognostic biomarkers. This is a clear example of how the implementation of quantitative approaches, either supervised or unsupervised, can represent a way to detect unseen patterns that hold predictive and prognostic value (43).

Multi-center, collaborative efforts can represent an asset by helping the collection of a huge amount of clinical and histological data to be analyzed through ML; large consortia such as European Reference Network for Rare Liver Diseases (ERN-RARE LIVER) or the International AIH Group can be leveraged to this end. Unsupervised techniques can be devised and validated in order to cluster individuals affected by AIH according to different prognostic trajectories.

As regards autoimmune cholangiopathies, histological samples suitable for analyses are limited in number. The diagnostic accuracy of PBC-specific autoantibodies has determined a progressive reduction in the number of liver biopsies performed (45); in PSC, liver biopsy is required only in atypical cases, since diagnosis and monitoring are performed by magnetic resonance cholangiopancreatography (MRCP) (46). Notwithstanding, PBC and PSC could potentially benefit from WSI together with ML. PBC and PSC are rare diseases with poorly understood pathogenesis; quantitative analysis of histological slides compared to healthy controls and/or other liver diseases such as metabolic liver disease or viral hepatitis could point toward zonal and cellular differences that are specific for these conditions. In this way, highlighting histological areas of potential interest, ML can be a tool to facilitate hypothesis generation of novel physiopathological models that can be subsequently dissected in the lab. Furthermore, the implementation of AI in the histological assessment of autoimmune cholangiopathies may be of aid in differential diagnosis with other chronic cholestatic syndromes. Of note, the definition of small duct PSC is still problematic and its distinction from intrahepatic genetic cholestasis or autoantibody-negative PBC is a challenge. Whether quantitative information included in histological slides of patients with small duct PSC is useful to predict the evolution toward a large duct form is still unknown, and it might be worth exploring in an integrated approach together with AI applications to radiology.

As regards biomarker discovery and risk stratification, non-invasive tools are not accurate enough to depict the full cholestatic picture, and the variety of inflammatory and fibrotic patterns, together with the cellular milieu of biliary regeneration, are not captured by transient elastography or routine liver enzymes (47, 48). This is not trivial; there is increasing evidence of the prognostic role of ductular reaction in these conditions (47). The alterations of the physiological architecture of the liver and biliary tree architecture pinpointed by ML could also represent novel biomarkers. Quantitative biomarkers of inflammation, biliary damage, or fibrosis can be discovered by means of AI and correlated with non-invasive biomarkers.

As regards disease classification and definition, unsupervised learning algorithms can be applied to detect sub-phenotypes that could prompt disease re-classification. The latter aspect is of particular interest for variant syndromes and for better characterization of the ductopenic variant of PBC.

Overall, the introduction of ML in this field of medicine may at minimum generate new hypotheses and identify novel biomarkers; yet, robust studies validated in several cohorts will be required to change also everyday clinical practice.

### 3.2 Applications in radiology

#### 3.2.1 Aims and applications

In the last decade, the remarkable progress in liver imaging techniques has helped to characterize several liver diseases from a qualitative and quantitative point of view. The foreseeable introduction of AI in medical practice together with the generation of a large amount of high-quality imaging data poses radiology as a key player in precision medicine (13, 49).

To this end, two groups of AI-based techniques can be mentioned: radiomics, which relies on ML, and DL systems (based on neural networks) (6). Radiomics has emerged as a high-throughput computing technique that enables extraction of large amounts of quantitative features from medical imaging, mainly computed tomography (CT), magnetic resonance imaging (MRI), and positron emission tomography (PET) (50). This vast amount of variables can be correlated with specific clinical outcomes of interest, providing far more information than those detectable by an experienced physician (51). The main difference between radiomics and DL (also known as deep radiomics) is the methodology of feature extraction. In “standard” radiomics, image analysis experts derive a list of mathematical equations that are applied to the image; in DL, convolutional neural networks are used for automatic extraction of features without the need of pre-defined programming (30).

We can speculate that the ultimate goal of both techniques is the combination of radiological data with clinical and laboratory data and potentially other -omics, to develop more accurate predictive models that incorporate a wider spectrum of disease-related features (52, 53).

#### 3.2.2 Methodology

The methodological process has been classically divided in distinct phases (51). The prerequisite is the collection of high-quality images with standardized imaging protocols to allow the repeatability and reproducibility of the analysis. In case of multicenter studies, as image resolution and intensity can be different depending on image acquisition and reconstruction procedure, preprocessing of the collected images is mandatory. This step is typically called *data acquisition and normalization*.

After preprocessing, one should define a region of interest (*segmentation of region of interest or ROI*). In most radiomics studies on liver diseases, the segmentation is performed by a radiologist as manual segmentation. Alternatively, the identification of ROI can be done by computer analysis through specific algorithms (automatic segmentation), with an optional input provided by the radiologist (semiautomatic segmentation). From the defined ROI, quantitative data are subsequently extracted (*feature extraction*). Informative data include both manual engineered features, like patterns of intensity, texture and shape, and abstract DL features. Of all the quantitative features extracted from the ROI, only the most informative will be retained, after feature selection through computational methods. Selected features are then fitted in a specific model of analysis. While a single modeling technique is often used, multiple-modeling methodology must be preferred to limit effects on prediction performance. Internal and possibly external validation of the model should be performed to avoid overfitting the model (*features selection, modeling, and validation*). The last and most important step of radiomics is to correlate the selected characteristics with the outcomes of interest to better characterize the disease (*image analysis*). Recently, the radiomics quality score (RQS) has been proposed to evaluate if a radiomic study matches with defined quality criteria in all steps (50, 51).

Overall, there are several methodological steps, and each of them needs high level of control to avoid biases in the prediction.

#### 3.2.3 Limitations

The first obstacle to the widespread application of radiomics in the study of liver diseases is the use of non-standardized image acquisition and reconstruction protocols even within the same institution, together with the need of a large amount of data, which is challenging and time-consuming. Moving forward through the radiomics workflow, the segmentation process has also some limitations. While manual segmentation is time-consuming, many automated and semi-automated algorithms are often suboptimal so that physicians are almost always needed to verify their accuracy. Moreover, in rare diseases such as AiLDs, automated segmentation algorithms do not exist. One solution might be the extraction of features through neural networks (54); the downside of this approach for AiLDs is the rarity of these conditions, while DL typically requires large datasets for training.

Another important issue that limits the broader adoption of AI algorithms is the lack of interpretability, which is the *black box* problem, namely, the difficulty of physicians to understand the predictions of ML algorithms (55) (see also *Section 3.5* for the debate about black box and explainable AI).

Overall, similarly to digital pathology, radiomics can also suffer from lack of standardization. It is conceivable that some strategies to address this issue can be shared between the two fields but most tasks are field-specific.

#### 3.2.4 Applications in AiLDs

Imaging plays a remarkable role in the diagnosis and management of PSC. Among medical imaging techniques, MRCP represents the main non-invasive imaging method for the diagnosis, risk stratification, and monitoring of patients with PSC (56). Yet, there is still lack of radiological features specific for PSC that allow exclusion of other causes of cholangiopathy. Thus, there is particular interest in the potential of quantitative imaging in terms of phenotypic characterization and differential diagnosis.

The highly variable disease course of PSC, likely associated with a variety of uncharacterized sub-phenotypes, represents a challenge for risk stratification. Several attempts have been made to develop reliable predictive tools in order to early discriminate patients with a more aggressive disease (56). To date, prognostic scores have been based mostly on laboratory and clinical data; some of them have been created by the application of ML algorithms, but without the inclusion of radiomic features (57, 58). Unfortunately, the fluctuating nature of serum markers of cholestasis during the course of the disease has hampered the accuracy of models based only on laboratory values so far. Liver stiffness measurements (LSMs) by either transient elastography or MRI elastography hold prognostic value (59, 60). Early arterial peribiliary hyperenhancement at MRI has been associated with higher Mayo risk scores and poorer prognosis (61). The ultrasound evaluation of incremented spleen size has also been correlated with major clinical outcomes in PSC (62).

The ultimate turning point in the evolution of radiological PSC characterization is the possibility to derive quantitative data from MRCP scans. There is mounting evidence on the accuracy of MRCP+, a novel image processing software that is able, through the creation of a 3D-enhanced model of the biliary tree, to provide quantitative metrics of the ductal anatomy, generating data on biliary tree volume, median diameter of the extrahepatic bile ducts, and number, length, and severity of strictures and dilatations (63). AI takes part in the segmenting, enhancing, and pre-processing of images together with the modeling of the derived information.

There is evidence supporting the reliability of MRCP+ metrics as a non-invasive tool to differentiate pediatric PSC from pediatric AIH (64, 65). In addition, MRCP+ parameters hold prognostic value, as proven by their strong correlation with validated biochemical and semi-quantitative MRCP-based risk scoring systems (66, 67). The application of AI on large-scale MRCP+-derived quantitative data has the potential to significantly improve the current diagnostic approach and prediction models of PSC.

As regards AIH, promising data employing Liver MultiScan technology have been recently presented. Following the evidence that multiparametric MRI (mpMRI) using iron-corrected T1 (cT1) relaxation maps provides an accurate, non-invasive quantitative biomarker of liver fibrosis and inflammation, recent works have shown that mpMRI, when applied to AIH patients, has a better performance in detecting residual disease activity than serological biomarkers (68, 69). Moreover, it seems that higher cT1 value at diagnosis correlates with a higher risk of loss of biochemical remission, gaining prognostic value (68, 69). Liver MultiScan technology seems to be a foreseeable accurate non-invasive biomarker in AIH that can enhance risk stratification. Multicenter prospective studies are needed to validate these preliminary findings, together with the implementation of AI platforms to leverage the large amount of data generated by these new technologies.

In conclusion, future implementations of radiomics and DL systems have the potential to improve our comprehension of the complexity of AiLDs. Looking forward, there are several potential future developments. Novel biomarkers can be investigated and validated; they may play a role as diagnostic, prognostic, and predictive tools to assist current clinical practice and clinical trials. Correlations of radiomic features with molecular parameters can enhance their contribution and potentially shed light on novel disease sub-phenotypes.

### 3.3 Population genetics

#### 3.3.1 Predicting phenotype based on genotype: A supervised learning approach

From a genetic perspective, AiLD are complex traits (70); in other words, their genetic architecture is not monogenic but dependent on the interplay of several genetic variants. The field of AiLD is still at the dawn of the big data era. While GWAS have been performed for each of the three conditions, whole-exome and whole-genome sequencing data are still missing. For all three conditions, single-nucleotide polymorphisms (SNPs) within the human leukocyte antigen region have a significant role in shaping their genetic risk (71–73); yet, several non-HLA variants have been described for PBC (74) and PSC (73), and more recently also for AIH (75). The discussion of the large topic of missing heritability is out of the scope of this review (76); however, it is worth mentioning that large portions of the heritability of AIH, PBC, and PSC are yet to be characterized (77). Whole-exome and whole-genome sequencing have clearly revealed that the identification of rare predisposing variants with large effect size is useful to fill this gap of knowledge (78, 79). Evidence in AiLD is scanty, mostly available for PSC, where autosomal-like patterns of inheritance have been identified in some families (80, 81), although it is likely that AiLDs derive in most cases from the interaction of some environmental triggers on the ground of a predisposing genetic background mostly composed of common variants. That said, the utility of PRSs, which are typically based on common variants, in AiLD is still a matter of debate (77).

In this paragraph, we focus our attention on the possible applications of ML on GWAS data, since they represent by far the largest data already available in this field.

ML is considered a complementary tool in population genetics, where several methodological hurdles need to be overcome. Research in population genetics has mostly focused on the formalization and validation of statistical models that describe patterns of variations and their application to experimental molecular data (82). While classical population genetics has been mainly characterized by parameter estimation in the context of a predetermined probabilistic model (typically the Wright–Fisher model), the target of ML is optimization of the accuracy of predictions (82). PRS predictions are based on a linear parametric regression model, with strict assumptions like additive effects, independent effects, normal distribution of the data, and independence of observations (83). These assumptions are often not valid in complex diseases like AiLDs. For example, thanks to their non-linearity, ML algorithms allow to account for complex interactive effects between associated alleles (84). Another peculiar and powerful feature of ML is its capacity to handle thousands of dependent variables, each characterized by a massive amount of information; this ability is of interest in the genomics world, where increasing dimensionality of data is an issue (82).

In population genetics, the output could be represented by the status (case or control) or a continuous phenotype (such as the value of a blood biomarker of interest), and the features are the individual sample genotype data (83). Data feature selection is the key step to obtain an accurate ML model (84). There are a few methods (embedded methods and wrappers) useful to select only informative SNPs as potential predictors (83).

The research question should be clear: does one want to predict outputs or to interpret data? The generative approach builds a model for two classes in a supervised manner, while the discriminative approach focuses only on separating them *via* an unsupervised approach.

The main application of supervised ML in population genetics is to build a model to classify cases and controls based on SNPs. This approach has the research aim to leverage AI to create a feature ranking of the most significant genetic variants that are inherently specific for the disease of interest and to create a polygenic model that can complement PRS. Moreover, ML can incorporate other relevant information such as sex to create hybrid models that can risk stratify the genetic liability already at birth. A caveat that should be carefully considered is that only “pure” phenotypes should be included as cases, to avoid confounding.

Another possible application of supervised ML in AiLDs could be to identify novel predictive features (SNPs) associated with phenotype, possibly looking at biologically distinct sub-phenotypes of the disease (early *vs* advanced disease, onset at younger age *vs* older age, positivity for specific autoantibodies). In this way, a predictive model is generated, taking advantage of the different contribution of variables within the training genotype data (83). After the training phase, the models with the maximum predictive power are selected for validation. This stage is essential to avoid overfitting and is usually achieved by cross-validation (dividing the original dataset into a training set and a test set). Nonetheless, external replication is still required for the final validation of the model (83).

Unsupervised approaches may be used to cluster patients according to genotype data and investigate whether these novel groups have different clinical presentations, trajectories, and treatment responses. Based on the availability of other omics, clustering can be extended to genomics and transcriptomic data for example. After generating clusters with hypothesis-free means, it is mandatory to understand if they hold biological and clinical significance, to create a classification that is really meaningful for clinicians. Yet, datasets having a different set of omics for the same group of individuals are seldom available. In addition, it is worth mentioning that, despite the growing body of GWAS data available in public repositories, there is still a large fraction of data that is not publicly shared with the community of researchers. Privacy issues do exist and the matter of balancing privacy rights with the need of sharing knowledge for the sake of the scientific advancement is a hot topic in genetics (85).

#### 3.4 Studying gene–gene interactions: A task for unsupervised learning

ML can also be helpful for studying epistasis, a so far neglected topic that may account for part of the missing heritability (86). Epistasis is difficult to study in humans compared to simpler animal models such as *Drosophila melanogaster*, and has many computational issues. Statistical definition of epistasis is that of interaction, the departure from a linear model describing how a number of predictors (x_i_) predict the outcome (the phenotype y). y can be a quantitative measure (e.g., height) or a binary outcome (case *vs*. control), so that linear or logistic models should be used, respectively.

If a locus of interest does have an influence on the phenotype and this happens *via* an interaction with another locus, it turns out that a model that incorporates interaction may increase the power to detect the effect of the locus of interest to the phenotype. For example, if the locus of interest is A, it may be more interesting to compare a model where the effects of locus A and B, and their interactions are included in a model where all terms (either main or interaction) involving locus A are removed.

Yet, in GWAS, several loci of interest should be investigated. The simple way is an exhaustive search of all possible pairs of loci studying interactions for each couple (two-locus interaction) or replicating the three degrees of freedom test iteratively. It is evident that a multiple testing issue arises and corrections should be employed, but this leads to the detection of only huge epistatic effects (87). Computational power (e.g., computer cluster) is required, but these analyses are still feasible; the real point is the lack of scalability to higher-order interactions. Pre-filtered loci that show some degree of significance in terms of correlation with the phenotype may be reasonable, but it is flawed by the exclusion from the analysis of those loci that do not show association with the phenotype (88).

One strategy is to guide the analysis based on biological plausibility (e.g., based on known interactions at the protein level, such as interactions among transcription factors and their targets, or proteins belonging to the same biological pathway). Another strategy is to use ML, which does not demand a marginal effect in place. ML can overcome the obstacles encountered by traditional regression-based methods since it works without a prespecified model and explores different models to search for the most computationally efficient one, avoiding a comprehensive research (89). From a mathematical point of view, ML does test for associations allowing interactions rather than testing directly for the interaction *per se* (see above). Another advantage is that there is a long line of research in computer science for problems like feature selection and data mining. For example, the Random forest method builds a tree-fashioned model measuring the effect of each SNP both individually and through interactions, generating a feature ranking, as compared to the list of *p*-values provided by PLINK, i.e., the gold standard software used in GWAS studies (89, 90). Since ML is not exhaustive and adopts heuristics, cross-validation and external validation steps are essential to avoid overfitting of the training set; if the sample size is small, parametric methods may be better suited for the analyses than ML techniques (91). An important caveat should be mentioned: epistasis can also be seen from a functional point of view (functional epistasis) rather than a statistical one. This means that epistasis occurs in biology and may be present despite lack of signals from quantitative studies (87, 88).

Overall, the investigation of gene–gene interactions has represented a methodological challenge for long. There is high expectation that AI-based pipelines can solve at least some issues, even though it is likely that large amounts of data will be required to fully recapitulate the network of gene–gene interactions.

### 3.5 Integrative multi-omics

ML represents a potent tool for analyses of data derived from high-throughput sequencing. As for other scientific fields, the possible applications of ML can be (1) generation of models for classification; (2) clustering of individuals in groups; and (3) feature selection. In this section, we provide several examples of these different applications.

The field of AiLD is still at the dawn of the big data era. Gene expression, proteomic, and metabolomic data come mainly from peripheral blood and single cohorts. More specifically, a recent study has shown that ML can discriminate between AIH and healthy controls based on gene expression profiles (92). A similar situation exists for microbiota, despite the growing interest on this side (93, 94). Single-cell data are becoming available for healthy liver in mice and human and in fibrotic livers (95); HCC and cholangiocarcinoma are also under investigation with these novel techniques.

Despite having a broad range of applications, ML is mostly useful for large datasets, such as those derived from microarray and high-throughput sequencing studies. Available data are genomic (SNP, whole exome, and whole genome), epigenetic (DNA methylation and histone modifications), transcriptomic (coding and non-coding RNAs, single-cell or bulk sequencing), proteomic, and metabolomic, with many others emerging (96). There is increasing availability of these datasets, which are frequently independently analyzed from different groups with different techniques: a brilliant example of such datasets is the UK Biobank (97). Datasets including the same set of omics for all individuals included in the cohort are rare. Most of the available omics data repositories were created without the vision of future multi-omics integration but rather to host data that were derived from a specific technology at a specific time. While linking different datasets is feasible, this is still extremely difficult, if not impossible, at the individual level. Cloud-based platforms for hosting several omics data for multi-omics integration have been developed (e.g., https://opendata.lifebit.ai/) (98). Despite shared guidelines have been produced (the FAIR Data Principles), the field is still lagging behind (99).

**Figure 2:**
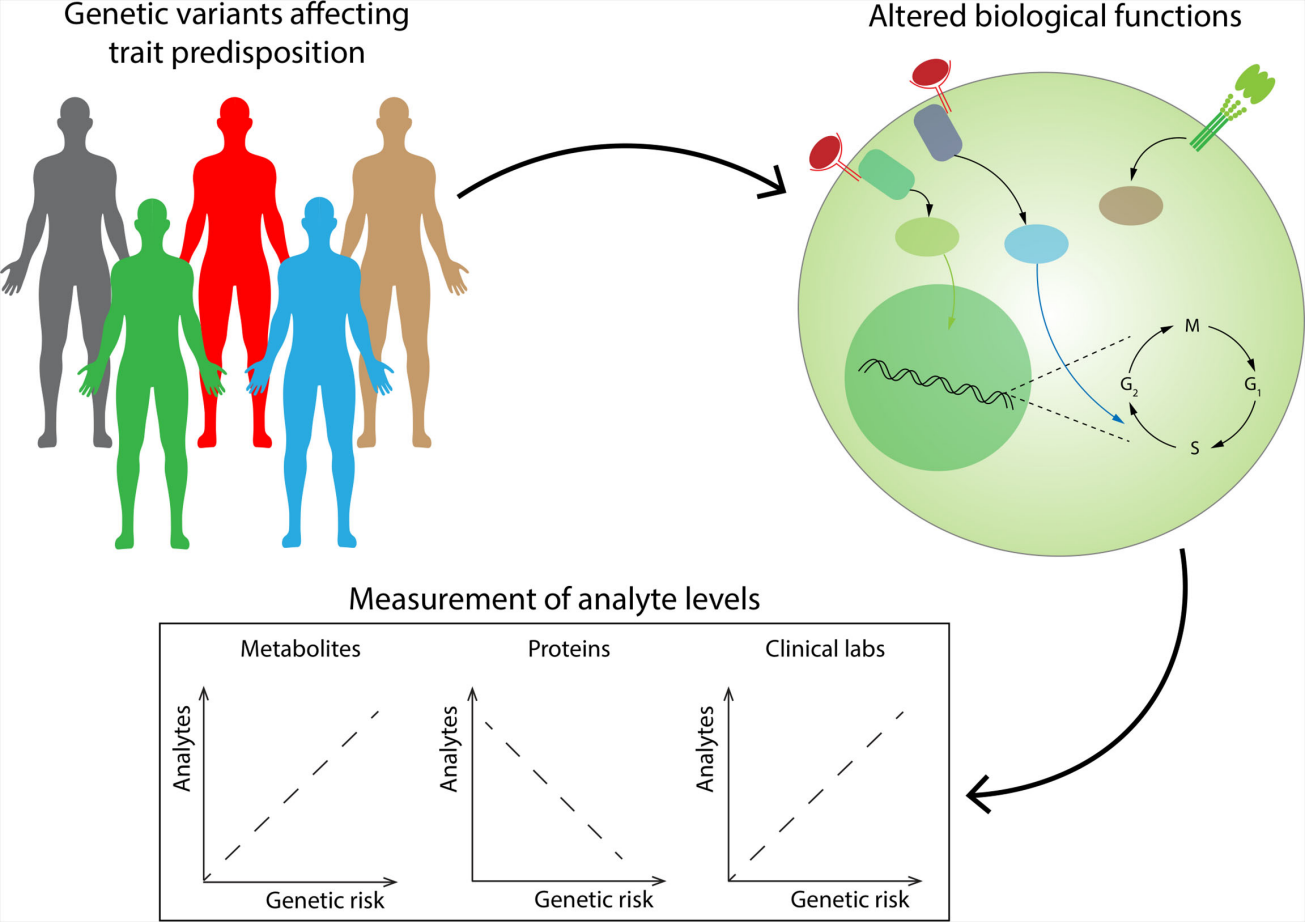
Healthy individuals with high genetic risk scores for a specific trait have already detectable abnormalities in several blood analytes. Genetic risk scores generated from risk variants for several traits have been associated with levels of plasma analytes (standard blood analytes, and proteomic and metabolic measurements), revealing that a nonnegligible level of dysregulation of these analytes can already be found in healthy subjects with high genetic risk. These approaches could be potentially leveraged for early detection of diseases.

Integrative multi-omics is a rapidly growing field within systems biology (96). Multi-omics integration is considered a cornerstone of precision medicine initiative (100). Putting together different types of information can be important for biomarker discovery and risk stratification as well as for pathogenesis and disease definitions. A promising example of this approach has been presented by Wainberg et al. (101) (**Figure 2**). The cohort under study was taken from the Arivale Scientific Wellness program, which included thousands of subjects undergoing several analyses: whole-genome sequencing or SNP microarray genotyping, and proteomic, metabolomic, and clinical laboratory measurements from 2015 to 2019. Authors generated polygenic risk scores (PRS) for 54 traits previously investigated through genome-wide association studies (GWAS) and investigated correlations between genetic risk scores and analytes, revealing that healthy subjects with high genetic risk show dysregulations of analytes that are similar to those found in disease. While some of them were expected (abnormalities in creatinine in patients with high genetic risk for chronic kidney disease), some other associations were novel (such as abnormalities in the metabolite 4-cholesten-3-one in patients with high genetic risk for PSC). Correlations were assessed by Glass’ Δ (a measure of effect size evaluating the difference in standard deviations among groups); would ML add value in a study with this design? Unsupervised learning through association mining would be probably worth pursuing and may reveal other interesting findings arising from data.

**Figure 3:**
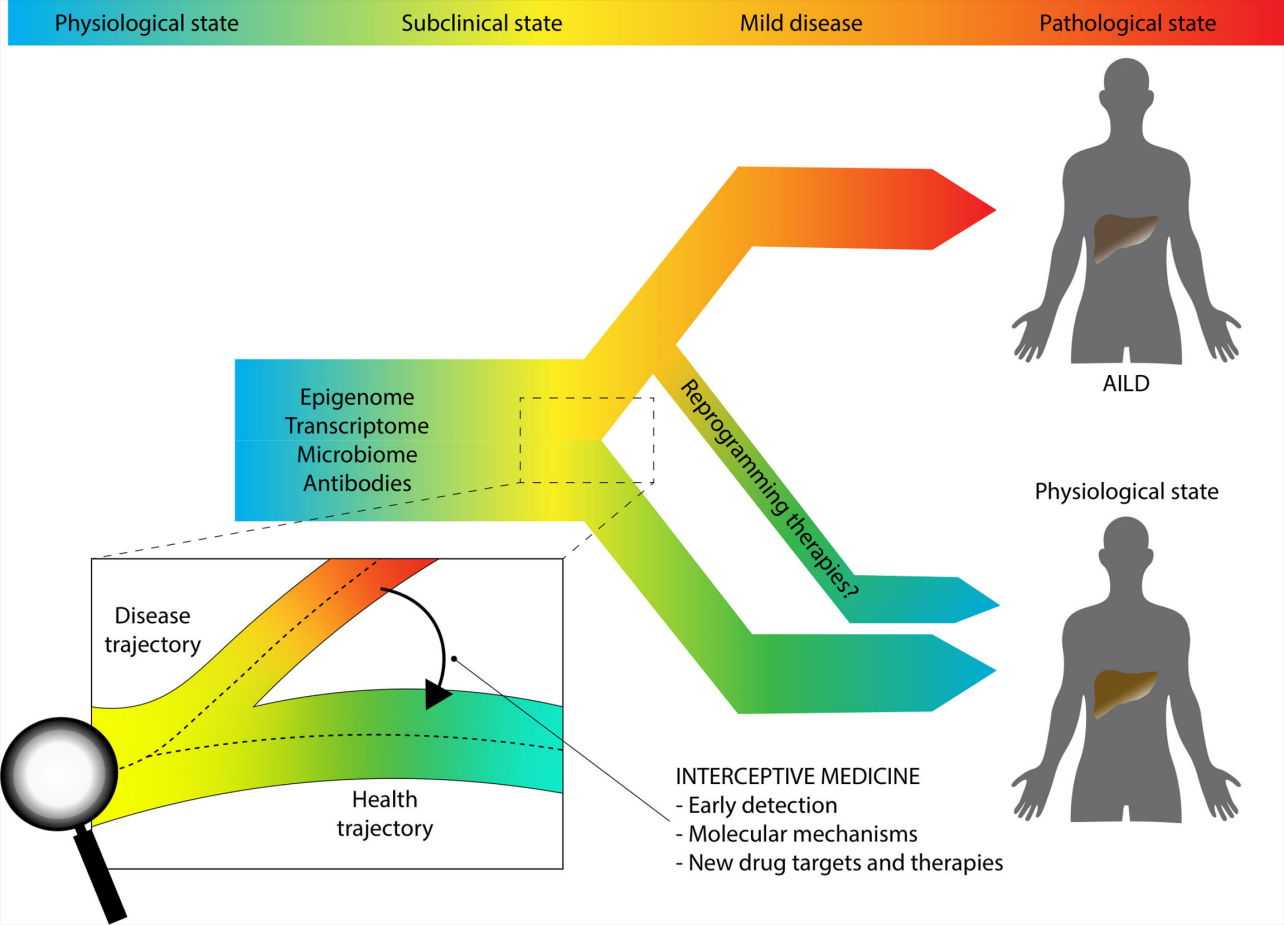
Interceptive medicine. The paradigm shift to study complex chronic immune-mediated traits proposed by the LifeTime Initiative and the SYSCID Consortium.

A much promising multi-omics approach is that described by the European LifeTime Initiative, which aims to integrate single-cell sequencing techniques, imaging, and patient-derived experimental disease models by means of AI (100). Investigators behind the LifeTime Initiative have introduced the concept of *interceptive medicine*, which is the early interception of disease based on more accurate cellular and molecular diagnostics. In other words, the idea behind interceptive medicine is to combine several breakthrough technologies such as single-cell sequencing and DL to track the process of the disease in an unprecedented way, highlighting potential druggable pathways that are significant in the early phase of the disease before the fibrotic process occurs (for inflammatory disease) or the disease spreads throughout the body (for cancer). (**Figure 3**). Chronic inflammatory diseases (CIDs) like AiLDs are often detected late when tissues have already undergone extensive and non-reversible changes, hindering therapeutic options. This can be due to several reasons. We lack longitudinal tracking of cellular heterogeneity and molecular cell trajectories from a healthy to a diseased state, there is often fragmentation of approaches without systematic profiling of patients, computational algorithms for integration are still under development, and drugs are still given to most patients without a clear insight into the precise molecular abnormalities within the specific subject. Despite adopting a systematic approach and involving thousands of subjects that underwent deep phenotyping, the previously mentioned study from Wainberg and colleagues still suffers from some of the issues raised by LifeTime investigators. For example, longitudinal analyte data were collapsed to their median values, because genetic risk does not change over time; transcriptomics profiling was not included, and single-cell technologies were also not employed; finally, no ML or other AI algorithms were used (101).

The LifeTime initiative aims to develop cutting-edge pipelines that incorporate different single-cell technologies, to investigate cellular heterogeneity, and spatial molecular information, to better define the location of disease cells within the tissue. This information will be collected longitudinally by small/liquid biopsies and integrated with EHRs of patients. Organoid models derived from healthy and diseased individuals will represent the experimental arm of the pipeline. It is self-evident that the amount of information generated will need dedicated bioinformatics skills and infrastructures and, ultimately, ML. One of the envisioned final goals is the adoption of AI-based systems that will aid clinical decisions in everyday practice. This project identified five pillars: cancer, neurological disease, infections, cardiovascular diseases, and CIDs. As regards the latter ones, where AiLDs fall within, the urgent target is the investigation of cellular heterogeneity and how this influences disease course and differences in treatment response.

A systematic approach devoted to CIDs has been proposed by the SYSCID (*a systems medicine approach to chronic inflammatory diseases*) consortium (102). The consortium acknowledges that the field of immune-mediated diseases is lagging behind cancer and cardiovascular areas in its shift toward precision medicine, due to some hurdles. The first one relates to the problem of missing heritability, the concept that the successful GWAS have identified many variants associated with the risk of developing CIDs but each with little impact *per se* (76); many strategies are currently suggested to fill this gap, including adoption of ML on top of classical statistical genetics (86). Yet, missing heritability does not affect only CIDs but most of the complex traits (76). A second issue is related to the fragmentation of diagnostic and therapeutic pathways for CIDs despite being characterized by overlap in their molecular risk map; SYSCID researchers advocate for the development of dedicated centers for inflammation medicine, where different specialists take care of these diseases, similarly to what has already occurred for cancer. The third issue is represented by the discrepancy between the complexity of omics data and the need for simple scores in clinical practice; this gap is still large for CIDs. Longitudinal tracking of what happens in diseased tissues is probably unfeasible, due to inaccessibility, calling in action blood biomarkers; to this end, the study of Wainberg et al. represents a good proxy for future endeavors. The cultural paradigm shift needed for the birth of System Immunology as a discipline is to move from a hypothesis-driven approach studying single molecules, single-cell types, etc. toward a hypothesis-free integration of different layers of information: this is where ML may play a key role thanks to its characteristics. There is evidence that heterogeneity occurs at the interindividual level [e.g., the identification of cell-type-specific molecular quantitative trait loci (QTLs) that are dependent on different genetic variants] and at the intraindividual level (thanks to the characterization of different populations of cells within tissues by employing single-cell sequencing). Like the LifeTime Initiative, SYSCID also works under the European research scheme of Horizon2020 and focuses on three paradigmatic autoimmune diseases: rheumatoid arthritis, systemic lupus erythematosus, and inflammatory bowel disease. Five layers of data will be available for approximately 50,000 individuals: SNP variants, DNA methylome, transcriptome, immunoglobulin glycome, and gut microbiome. Canonical statistical modeling and novel ML techniques will be used to identify biomarkers, subtypes of disease, predictive models for tailored treatments, and novel reprogramming strategies.

Supervised learning could be used to separate groups according to clinical parameters of interests, such as treatment response; this will be likely a complementary or subsequent approach, since it requires a certain pre-test hypothesis. Unsupervised learning will be crucial in aggregating individuals based on the different layers of information. Kobak et al. have recently described pitfalls that may possibly occur in single-cell RNA sequencing data analysis (103); the addition of multiple layers of information will add even further complexity to data reduction algorithms. Multi-scale models will potentially enable to predict disease phenotypes at the cellular level (100).

To summarize, making multi-omics integration a process that generates valuable scientific knowledge to translate in the clinic requires the collaboration among several scientists from different fields (4). Yet, the potential output of such an approach is potentially disruptive for the field of immune-mediated diseases and AiLDs more specifically.

### 3.6 Opening the black box: The importance of explainable AI

One of the fathers of the DL paradigm, Yoshua Bengio, recently highlighted, in a keynote speech at the IEEE World Congress on Computational Intelligence, how DL, for the time being, has been good in dealing with *a subset* of relevant activities. According to Bengio, the subset is related to the realm of *intuition*, rather than *explanation*: algorithms may perform well, but still struggle in *explaining why they perform well*.

Following Daniel Kahneman’s classification of human intelligence (104), Bengio asserts that ML already achieved good results in emulating the behavior of what Kahneman refers to as *System 1* (which leads to intuitive unconscious decisions for human beings), while its path towards the emulation of *System 2* (that leads humans to deliberate and make conscious decisions) is still ongoing.

This is why many researchers focus on the development and on the extension of rule-based modeling techniques, so that each prediction is motivated by the rule(s) determining it. This is the case, for instance, of decision trees (105) and the logic learning machine (106). Considering the importance of keeping clinical experts at the core of the decision process in medicine, this kind of approach, focused on *interpretability*, may be of particular interest in the field.

DL has outstanding potential, but it is challenging to interpret DL systems and translate them in clinical practice. The lack of explainability makes it difficult to identify and tackle biases present in training sets. Most importantly, the research aim should be clear. If the goal is automation and technological aid to humans, lack of interpretability is probably much less important. Yet, when models are devised to make predictions that can change medical decisions, it is ethically difficult to accept a model that cannot be tracked in its work (107, 108). Furthermore, trustability is likely to be proportional to interpretability: it is less likely that a clinician would trust a model if he/she is not able to understand even a tiny part of it.

The encounter between ML and the biomedical field forces both specialists to learn something that is not typical of their domain (109). If clinicians will progressively be surrounded by concepts related to ML and its applications, ML scientists should work to fill some gaps that are still present in the ML literature (4). Many core statistical concepts, like calibration of estimates, confidence of estimates, or power calculation, have not been incorporated in ML models.

Overall, we acknowledge that these statements could be outpaced by changes in the field, which is moving rapidly and may pose new challenges, making these ones rapidly outdated.

## 4 Hurdles, limitations, and pitfalls

High expectations behind AI do exist (13); yet, we are progressively learning that several issues have to be tackled before full clinical implementation is possible (109). We can divide these issues into those concerning model development and those regarding model deployment (6, 110). It is also important to mention the difficulties related with the de-identification process required by data protection laws, especially relevant for the genetic field (85).

We mentioned the need for data standardization and explainability of models. It is also worth mentioning the issue of having a diverse dataset to have more generalized models; similarly to what is known in population genetics, where most of GWAS have been performed in populations of European origin, under-representing most of the other ancestries (111), there is a risk for training models in homogeneous populations where demographic factors are specific and, importantly, the etiology of the liver disease is different. To make an example, a model for non-invasive prediction of liver fibrosis trained in a US-derived cohort, where non-alcoholic fatty liver disease is the most prevalent cause, would probably fall short when applied in a Taiwanese cohort where chronic hepatitis B is leading. The need for diversity poses even a greater challenge for rare diseases like AiLDs, stressing the importance of creating big multicenter consortia and collaborative efforts to collect large-scale data.

Reproducibility of ML studies is a hot topic. There is evidence that the majority of studies do not follow a rigorous and consistent methodology (112, 113). Researchers in the field of computer science applied to medicine have been developing guidelines and standard methods that should be followed (114).

Yet, the field should also start focusing more on clinical deployment rather than doing only retrospective analysis for validation purposes (110). Digital transition will require building new infrastructures, training healthcare workforce, and involving patients in this process. Wherever possible, AI-based models should aim toward liberating the healthcare personnel from repetitive tasks to have more time to spend with their patients (13). Novel data sharing technologies are also required, to balance the need for data protection and to offer at the same time a large amount of data to train the algorithms. Blockchain-based swarm learning seems to offer a quite promising approach to this end (115, 116).

While AiLDs will share general hurdles for the implementation of AI in medicine, there are also disease-specific obstacles. We believe that AiLDs share with other rare conditions the difficulty in having sufficient sample sizes for these data-hungry new methodologies; despite the birth of worldwide consortia, the field will never reach the numbers of cardiovascular or cancer specialties. The wide heterogeneity of phenotypes seen in the clinic represent another peculiar obstacle for supervised approaches, which require clear-cut phenotypes as outputs. On the other hand, unsupervised learning techniques, although much awaited in AiLD for their capacity to detect patterns within data and pinpoint sub-phenotypes that are only intuitively noticed by clinicians, need bigger samples than supervised ones.

Overall, we are at a unique juncture in the history of medicine, with new technological avenues bringing together new challenges.

## 5 Conclusions

**Figure 4:**
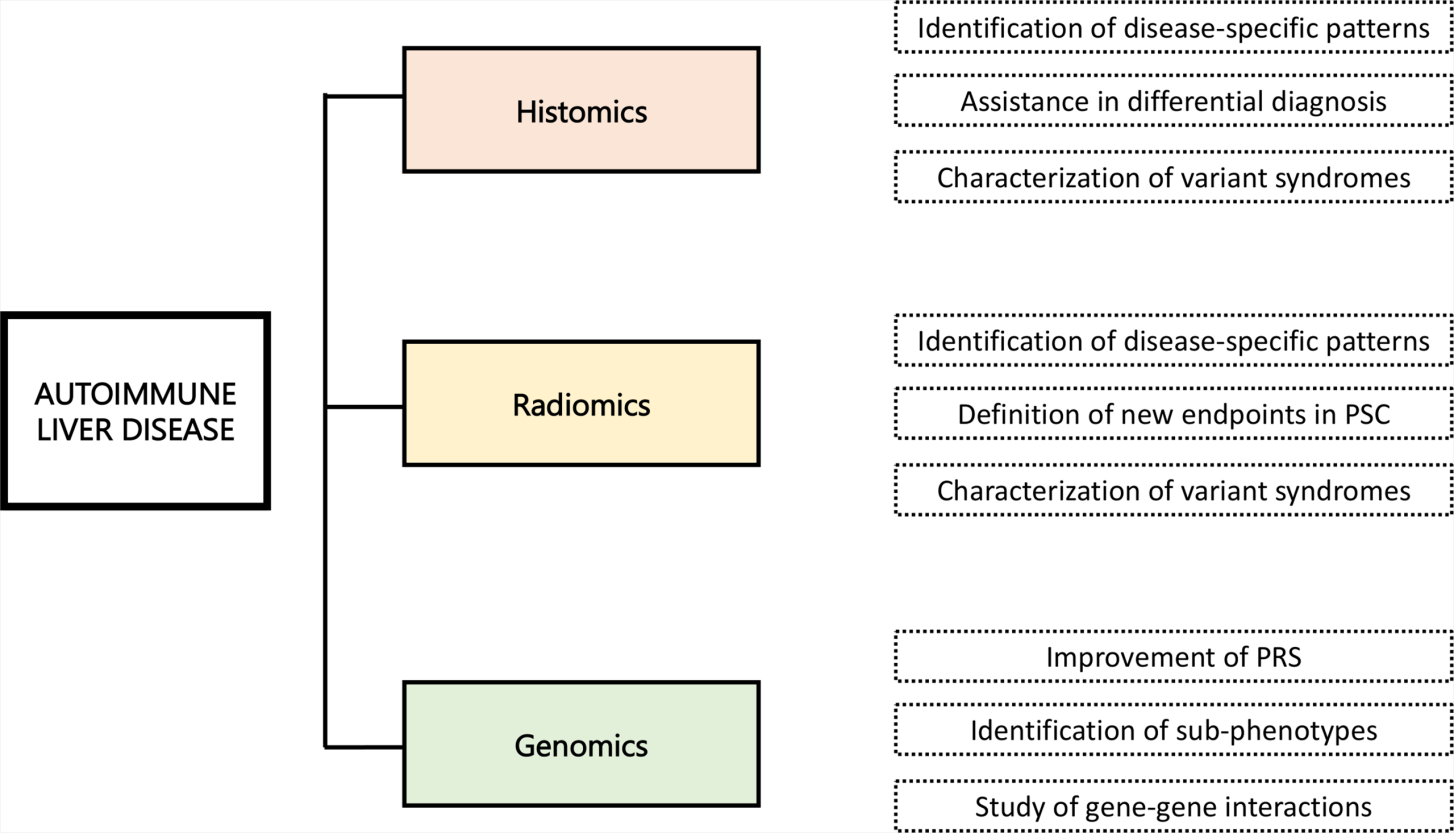
Future applications of artificial intelligence in the field of autoimmune liver disease.

The application of ML to big data in medicine, and more specifically in AiLDs, is challenging. **Figure 4** recapitulates the future applications of AI in the field of AILD. Major scientific, infrastructural, and cultural changes are needed (102). International endeavors should be implemented, to cut costs and address many, if not all, immune-mediated diseases altogether, breaking cultural barriers between clinicians and computational scientists, and educating the public about the benefit of the access to big personal health datasets rather than focusing on the privacy issues. It is essential to cut down the barriers to accessing genomic data derived from direct-to-consumer testing, such as 23andMe and other initiatives, which have been largely underutilized so far (117). If it is true that rare diseases suffer from minor availability of large amount of data, it would be of great value to take advantage of data generated by wearable devices and mobile phones, or from genetic analyses that individuals perform without clinical indication (e.g., 23andMe and others). Moreover, many data regarding rare conditions are already present in public repositories but may be scattered among different platforms and datasets.

Explainability of AI will be probably required by regulatory agencies to support systemic approaches and to accept their future translation into clinical protocols and pathways (118). Another caveat is that ML mostly finds correlation and is a hypothesis-generating tool; this means that it can open new doors, but all new ideas should still be tested experimentally, either in the laboratory or in clinical trials.

Nevertheless, despite all these potential obstacles to the application of AI in biomedicine, we should realize that this process could revolutionize the way we diagnose and treat our patients, which is the ultimate goal of translational research. Successful implementation will require investment in healthcare workforce education and technological infrastructures, together with involvement of patients and the creation of a culture of innovation and learning. To do so, we advocate that health services together with regulatory bodies should create a robust framework able to leverage the opportunities and address all the challenges that AI provides.

## Author contributions

The first draft of the manuscript was prepared by AG with the support of MS, LC, and DV. CM has created and processed all figures. All authors contributed to manuscript revision, and read and approved the submitted version.

## Funding

This research was partially supported by the Italian Ministry of University and Research (MIUR) - Department of Excellence project PREMIA (PREcision MedIcine Approach: bringing biomarker research to clinic).

## Acknowledgments

Alessio Gerussi, Miki Scaravaglio, Laura Cristoferi, Pietro Invernizzi, and Marco Carbone are members of the European Reference Network on Hepatological Diseases (ERN RARE-LIVER). This work has been supported by grants of the Italian Ministry of Health in the role of auto-reactive hepatic natural killer cells in the pathogenesis of primary biliary cholangitis (PE-2016-02363915) and in the biocompatible nano-assemblies to increase the safety and the efficacy of steroid treatment against liver inflammation (GR-2018-12367794). The authors thank AMAF Monza ONLUS and AIRCS for the unrestricted research funding.

## Conflict of interest

Author DV is employed by Rulex.

The remaining authors declare that the research was conducted in the absence of any commercial or financial relationships that could be construed as a potential conflict of interest.

## Publisher’s note

All claims expressed in this article are solely those of the authors and do not necessarily represent those of their affiliated organizations, or those of the publisher, the editors and the reviewers. Any product that may be evaluated in this article, or claim that may be made by its manufacturer, is not guaranteed or endorsed by the publisher.
